# Genome-wide methylation profiling differentiates benign from aggressive and metastatic pituitary neuroendocrine tumors

**DOI:** 10.1007/s00401-024-02836-5

**Published:** 2024-11-23

**Authors:** Jelena Jotanovic, Henning Bünsow Boldt, Mark Burton, Marianne Skovsager Andersen, Daniel Bengtsson, Thomas Olsson Bontell, Bertil Ekman, Britt Edén Engström, Ulla Feldt-Rasmussen, Ansgar Heck, Antonia Jakovcevic, Jens Otto L. Jørgensen, Ivana Kraljevic, Jacek Kunicki, John R. Lindsay, Marco Losa, Paul Benjamin Loughrey, Dominique Maiter, Maria Maksymowicz, Emilija Manojlovic-Gacic, Jens Pahnke, Stephan Petersenn, Maria Petersson, Vera Popovic, Oskar Ragnarsson, Åse Krogh Rasmussen, Zita Reisz, Wolfgang Saeger, Camilla Schalin-Jäntti, David Scheie, Maria Rosa Terreni, Olli Tynninen, Ben Whitelaw, Pia Burman, Olivera Casar-Borota

**Affiliations:** 1 Department of Immunology, Genetics and Pathology, Uppsala University, Uppsala, Sweden; 2 Department of Clinical Pathology, Uppsala University Hospital, Uppsala, Sweden; 3 Department of Clinical Research, University of Southern Denmark, Odense, Denmark; 4 Department of Pathology, Odense University Hospital, Odense, Denmark; 5 Department of Clinical Research, Faculty of Health Sciences, University of Southern Denmark, Odense, Denmark; 6 Department of Clinical Genetics, Odense University Hospital, Odense, Denmark; 7 Clinical Genome Center, University of Southern Denmark, Odense, Denmark; 8 Department of Endocrinology, Odense University Hospital, Odense, Denmark; 9 Department of Health, Medicine and Caring Sciences, Linköping University, Linköping, Sweden; 10 Department of Internal Medicine, Kalmar County Hospital, Kalmar, Sweden; 11 Department of Physiology, Institute of Neuroscience and Physiology, Sahlgrenska Academy, University of Gothenburg, Gothenburg, Sweden; 12 Department of Clinical Pathology, Sahlgrenska University Hospital, Gothenburg, Sweden; 13 Department of Endocrinology in Linköping, Department of Internal Medicine in Norrköping, and Department of Health, Medicine and Caring Sciences, Linköping University, Linköping, Sweden; 14 Department of Medical Sciences, Endocrinology and Mineral Metabolism, Uppsala University, Uppsala, Sweden; 15 Department of Endocrinology and Diabetes, Uppsala University Hospital, Uppsala, Sweden; 16 Department of Medical Endocrinology and Metabolism, Rigshospitalet, Copenhagen, Denmark; 17 Institute of Clinical Medicine, Faculty of Health Research Sciences, Copenhagen University, Copenhagen, Denmark; 18 Section for Specialized Endocrinology, Oslo University Hospital, Oslo, Norway; 19 Department of Pathology and Cytology, University Hospital Center Zagreb, Zagreb, Croatia; 20 School of Medicine, University of Zagreb, Zagreb, Croatia; 21 Department of Endocrinology and Internal Medicine, Aarhus University Hospital, Aarhus, Denmark; 22 Department of Endocrinology, University Hospital Center Zagreb, Zagreb, Croatia; 23 Department of Neurosurgery, Maria Sklodowska-Curie National Research Institute of Oncology, Warsaw, Poland; 24 Regional Centre for Endocrinology and Diabetes, Royal Victoria Hospital, Belfast Health and Social Care Trust, Belfast, UK; 25 Department of Neurosurgery, IRCCS San Raffaele Scientific Institute, Vita-Salute University, Milan, Italy; 26 Patrick G Johnston Centre for Cancer Research, Queen’s University Belfast, Belfast, UK; 27 Department of Endocrinology and Nutrition, UCL, Cliniques Universitaires Saint-Luc, Brussels, Belgium; 28 Department of Cancer Pathomorphology, Maria Sklodowska-Curie National Research Institute of Oncology, Warsaw, Poland; 29 Institute of Pathology, Faculty of Medicine, University of Belgrade, Belgrade, Serbia; 30 Translational Neurodegeneration Research and Neuropathology Lab, Department of Clinical Medicine, Medical Faculty, University of Oslo, Oslo, Norway; 31 Section of Neuropathology Research, Department of Pathology, Clinics for Laboratory Medicine, Oslo University Hospital, Oslo, Norway; 32 ENDOC Center for Endocrine Tumors, Hamburg, Germany; 33 University of Duisburg-Essen, Essen, Germany; 34 Department of Endocrinology, Karolinska University Hospital, Stockholm, Sweden; 35 Department of Molecular Medicine and Surgery, Karolinska Institutet, Stockholm, Sweden; 36 Medical Faculty, University of Belgrade, Belgrade, Serbia; 37 Department of Endocrinology, Sahlgrenska University Hospital, Gothenburg, Sweden; 38 Department of Internal Medicine and Clinical Nutrition, Institute of Medicine, Sahlgrenska Academy, University of Gothenburg, Gothenburg, Sweden; 39 Wallenberg Centre for Molecular and Translational Medicine, Institute of Medicine, University of Gothenburg, Gothenburg, Sweden; 40 Department of Nephrology and Endocrinology, Rigshospitalet, Copenhagen University Hospital, Copenhagen, Denmark; 41 Department of Clinical Neuropathology, King’s College Hospital, NHS Foundation Trust, London, UK; 42 Institute of Neuropathology, University Medical Center Hamburg-Eppendorf, Hamburg, Germany; 43 Institute of Pathology, University Medical Center Hamburg-Eppendorf, Hamburg, Germany; 44 Endocrinology, Abdominal Center, Helsinki University Hospital, Helsinki, Finland; 45 University of Helsinki, ENDO-ERN (European Reference Network On Rare Endocrine Conditions), Helsinki, Finland; 46 Department of Pathology, Rigshospitalet, Copenhagen University Hospital, Copenhagen, Denmark; 47 Department of Clinical Medicine, University of Copenhagen, Copenhagen, Denmark; 48 Department of Pathology, IRCCS San Raffaele Scientific Institute, Vita-Salute University, Milan, Italy; 49 Department of Pathology, Helsinki University Hospital, University of Helsinki, Helsinki, Finland; 50 Department of Endocrinology, King’s College Hospital, NHS Foundation Trust, London, UK; 51 Department of Endocrinology, Skåne University Hospital, Lund University, Malmö, Sweden

**Keywords:** Aggressive pituitary tumor, Pituitary carcinoma, Pituitary neuroendocrine tumor, Methylation analysis, Pituitary adenoma

## Abstract

Aggressive pituitary neuroendocrine tumors (PitNETs)/adenomas are characterized by progressive growth despite surgery and all standard medical therapies and radiotherapy. A subset will metastasize to the brain and/or distant locations and are termed metastatic PitNETs (pituitary carcinomas). Studies of potential prognostic markers have been limited due to the rarity of these tumors. A few recurrent somatic mutations have been identified, and epigenetic alterations and chromosomal rearrangements have not been explored in larger cohorts of aggressive and metastatic PitNETs. In this study, we performed genome-wide methylation analysis, including copy-number variation (CNV) calculations, on tumor tissue specimens from a large international cohort of 64 patients with aggressive (48) and metastatic (16) pituitary tumors. Twelve patients with non-invasive pituitary tumors (Knosp 0–2) exhibiting an indolent course over a 5 year follow-up served as controls. In an unsupervised hierarchical cluster analysis, aggressive/metastatic PitNETs clustered separately from benign pituitary tumors, and, when only specimens from the first surgery were analyzed, three separate clusters were identified: aggressive, metastatic, and benign PitNETs. Numerous CNV events affecting chromosomal arms and whole chromosomes were frequent in aggressive and metastatic, whereas benign tumors had normal chromosomal copy numbers with only few alterations. Genome-wide methylation analysis revealed different CNV profiles and a clear separation between aggressive/metastatic and benign pituitary tumors, potentially providing biomarkers for identification of these tumors with a worse prognosis at the time of first surgery. The data may refine follow-up routines and contribute to the timely introduction of adjuvant therapy in patients harboring, or at risk of developing, aggressive or metastatic pituitary tumors.

**Supplementary Information:**

The online version contains supplementary material available at 10.1007/s00401-024-02836-5.

## Introduction

Pituitary neuroendocrine tumors (PitNETs)/adenomas [48] are generally benign, with a favorable prognosis and satisfactory response to surgical and/or pharmacological therapy. Radiologically invasive tumors with unusually fast growth and/or significant progression despite standard treatments, including surgery, radiotherapy, and medical therapy, constitute < 1% of PitNETs [4, 10]. These tumors are classified as aggressive pituitary tumors (APT) according to the definition by the European Society of Endocrinology (ESE) guidelines [33]. Metastatic PitNETs, or pituitary carcinomas (PC), account for 0.1–0.6% of all surgically treated pituitary tumors and are defined by the presence of metastatic spread to the central nervous system and/or to distant locations [35, 48]. For simplicity, we will use the abbreviations APT for aggressive PitNETs without metastases, and PC for metastatic PitNETs throughout the manuscript.

There are currently no reliable histological markers to predict aggressive and metastatic behavior in early stage PitNETs, specifically before a clinically aggressive phenotype becomes apparent, i.e., prior to progressive growth despite repeated surgeries, medical treatments, and radiotherapy. Many benign pituitary tumors with elevated proliferation markers are well controlled with standard treatments. In contrast, the Ki67 index is low (≤ 3%) in approximately 20% of aggressive PitNETs [4]. Therefore, in clinical practice, a watchful waiting is often adopted, with a risk of delaying growth arresting therapy for the aggressive tumors.

The mutation rate in PitNETs is generally low with approximately 60% of benign tumors demonstrating no detectable genetic alterations [3, 5, 41]. Somatic mutations are more frequent in APT/PC [4], most commonly affecting the *ATRX* and *TP53* genes, especially in a subset of corticotroph tumors [8, 9, 16, 46], and the *SF3B1* gene in a proportion of lactotroph APT/PC [22, 40]. Since studies conducted on single cases or small series of sporadic APT/PCs have shown a few recurrent mutations in oncogenes and tumor suppressor genes, other molecular alterations, such as epigenetic events that regulate gene expression, are likely to contribute to tumorigenesis.

One of the critical phenomena in carcinogenesis is DNA methylation, an epigenetic modification of DNA that involves covalent binding of methyl groups to the 5-C end of cytosine, primarily on sequences containing CpG dinucleotides. Global DNA hypermethylation with subsequent silencing of tumor suppressor genes and DNA hypomethylation-related activation of proto-oncogenes are considered fundamental events in carcinogenesis [12, 19, 25]. Furthermore, copy-number variations (CNVs) are structural genome rearrangements and chromosomal regions gains or losses that, in addition to having a proposed pathophysiological and physiological role [31, 38], can serve as tumor biomarkers [6, 7]. Epigenetic events related to the acquisition of aggressive properties and metastatic potential in PitNETs remain largely unknown.

In the present study, we performed a genome-wide DNA methylation analysis on a large international cohort of aggressive pituitary tumors, both with and without metastases. Our goal was to explore whether aggressive and metastatic PitNETs cluster separately and demonstrate copy-number alterations that differ from those of benign tumors.

## Materials and methods

### Patient cohort

Sixty-five patients (40 males), including 48 with APT and 17 with PC, who were diagnosed and treated at specialized tertiary pituitary centers in 11 European countries (Belgium, Croatia, Denmark, Finland, Germany, Italy, Norway, Poland, Serbia, Sweden, and the UK) were enrolled in the study. A subset of 28 patients enrolled in this study was included in our previous study on *ATRX* gene expression in pituitary tumors [8]; no data on epigenetic alterations in any of the patients included in the present cohort have been published previously. The inclusion criteria were adult age (> 18 years) and strictly fulfilling the criteria for APT according to the ESE guidelines [33]: invasive tumor with unusually fast growth and/or clinically significant tumor progression despite surgery, radiotherapy, and standard medical therapy. In the PC group, seven patients presented with metastases within the central nervous system, seven had metastases to distant locations, and three had metastases in both the central nervous system and other sites. None of the patients had known metastases at the time of their first pituitary surgery. One patient was excluded as an outlier, as detailed below, resulting in a final cohort of 64 patients (39 males), comprising 48 with APT and 16 with PC.

The tumors were clinically classified based on the symptoms and results of the laboratory tests. Of the 64 APT/PC, 14 were clinically non-functioning. Among the 50 hormone-producing APT/PC, corticotroph and lactotroph tumors were the most common. Four patients presented with tumors with functional status that changed during the course of the disease; three silent corticotroph tumors developed into ACTH-secreting tumors, and one prolactin-secreting tumor changed to a growth hormone-secreting tumor. These patients were categorized into the Cushing’s and acromegaly groups, respectively. The specimens analyzed were obtained from surgeries performed before the phenotype changes.

A group of 12 patients (six males) who underwent surgery for a non-invasive PitNET (Knosp grades 0–2) at the Department of Neurosurgery, Uppsala University Hospital, was included for comparison. These patients had no signs of tumor regrowth or hormonal hypersecretion during ≥ 5 years of follow-up after surgery with or without standard pharmacological therapy. In the histological specimens, there was only slight cell atypia, < 2 mitoses per 10 high power fields (HPF; 1 HPF corresponding to 0.238 mm^2^), a Ki67 index < 3% and p53 expression in < 10 cells per 10 HPF.

**Fig. 1:**
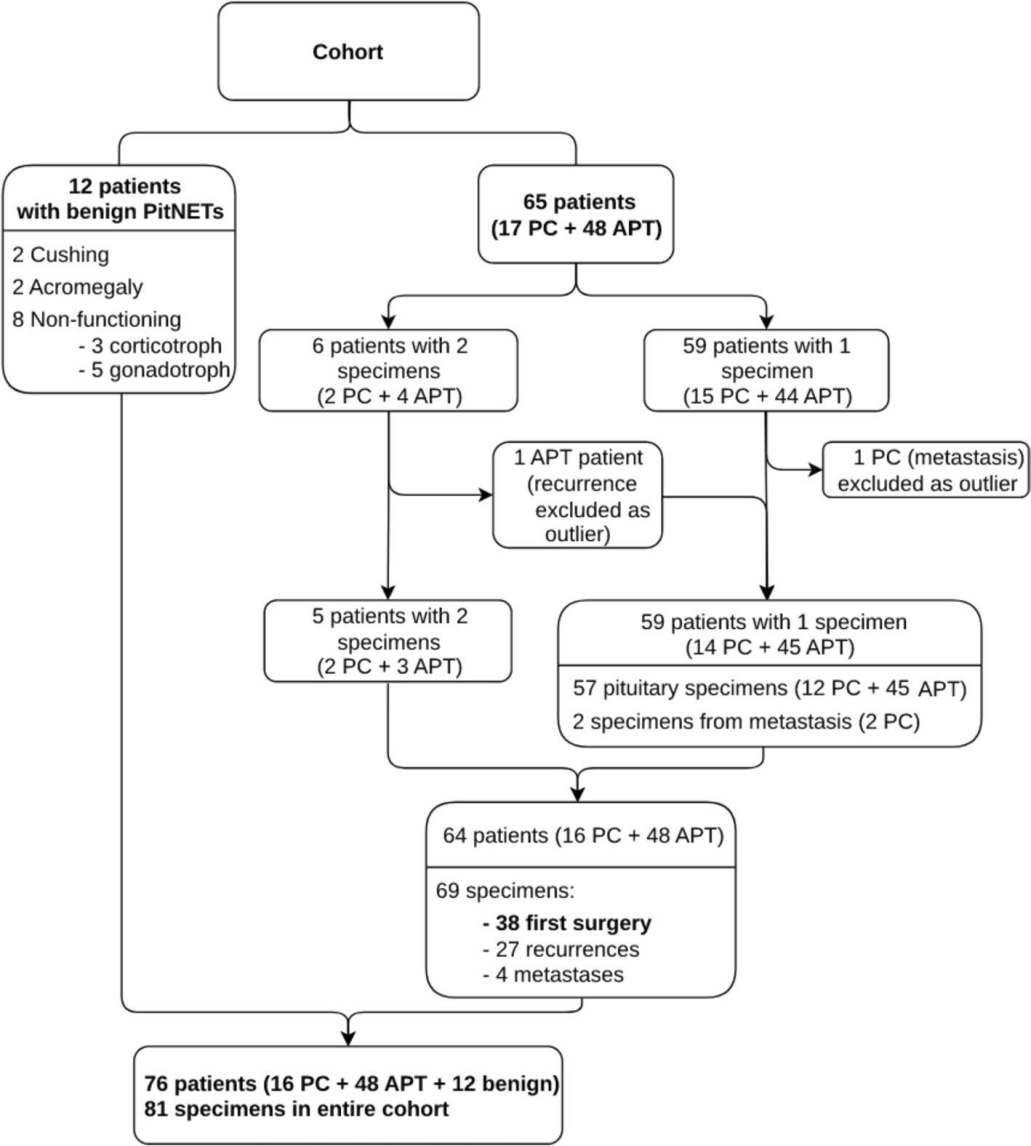
Chart showing the structure of the APT/PC cohort and the benign tumors

The distribution of the histological and clinical tumor types in the cohort is presented in Table 1. Details regarding the origin of all histological specimens analyzed in the study, including both APT/PC and benign tumors, are outlined in Fig. 1.

**Table 1:** Overview over the entire cohort

	Total		APT		PC		Benign	
	76		48		16		12	
Male *n* (%)	45 (59)		29 (60)		10 (62)		6 (50)	
F:NF	54:22		36:12		14:2		4:8	
Histological type (total, F:NF)								
Corticotroph	33	27:6	20	17:3	8	8:0	5	2:3
Lactotroph	16	16:0	12	12:0	4	4:0	0	0
Somatotroph	9	6:3	4	3:1	3	1:2	2	2:0
Somato/lactotroph	3	2:1	2	1:1	1	1:0	0	0
Thyrotroph	2	2:0	2	2:0	0	0	0	0
Gonadotroph	6	0:6	1	0:1	0	0	5	0:5
Silent PIT1	4	0:4	4	0:4	0	0	0	0
Plurihormonal^*a*^	3	1:2	3	1:2	0	0	0	0
Clinical type								
Cushing	27		17		8		2	
Prolactinoma	16		12		4		0	
Acromegaly	8		4		2		2	
TSH-oma	2		2		0		0	
TSH/FSH	1		1		0		0	
NF	22		12		2		8	

*F* functioning, *NF* non-functioning, *APT* aggressive pituitary tumor, *PC* pituitary carcinoma, *TSH* thyroid stimulating, *FSH* folliculostimulating hormone, *PIT*1 pituitary transcription factor 1, *SF*1 steroidogenic factor-1, *PRL* prolactin, *GH* growth hormone

^*a*^Two SF1 + PIT1 (one silent and one functioning TSH/FSH), and one silent SF1 + TPIT. The transcription factors SF1 and PIT1 respective TPIT were expressed in different subsets of tumor cells and not co-expressed

In the first surgery cohort (38 APT/PC and 12 patients with benign PitNETs), information on tumor invasion as assessed by magnetic resonance imaging (MRI) was available for 47 patients.

The Swedish Ethical Review Authority approved the study protocol, Dnr 2021-00073. PitNET tissue specimens from patients who underwent surgery at Uppsala University Hospital were partly obtained through the U-CAN project (www.u-can.uu.se) [13] and used in accordance with the ethical permission Dnr 2018–053.

### Tissue specimens

Formalin-fixed paraffin-embedded (FFPE) tumor tissue specimens were collected from the pathology departments at the participating centers. Hematoxylin–eosin-stained slides from all tissue specimens were reviewed by two pathologists (OC-B and JJ) to confirm the presence of representative tumor tissue and assess the fraction of viable tumor cells. In cases where a tissue sample contained both neoplastic and non-neoplastic pituitary tissue, the tumor tissue was macro-dissected for DNA extraction.

Tumor tissue specimens from multiple operations were available from 39 patients. When possible, specimens with the most representative and well-preserved tumor tissue from an early surgery, before radiotherapy or any pharmacological therapy, were selected for methylation profiling when possible. The first surgery specimens were excluded if they were too small or if the tissue was damaged (e.g., due to bleeding, ischemic damage, or damage from diathermia). In some cases, specimens could not be obtained. In five patients, for whom the intervals between pituitary surgeries were longer than 9 years or for whom material from the pituitary surgery as well as from the metastasis was available, two tumor tissue specimens were examined. This resulted in the analysis of a total of 69 APT/PC specimens (Fig. 1). Among the 16 patients with pituitary carcinoma, tissues from the first surgery were examined in seven cases, while tissues from recurrences were analyzed in seven cases (two of whom had paired specimens from pituitary surgery and metastasis). In two patients, tissues were only available from metastases. Seven of the nine patients with PC had received adjuvant treatment, including radiotherapy (*n* = 3), or radiotherapy and temozolomide (*n* = 4; one of these also received bevacizumab) before the specimens from recurrence/metastasis were obtained.

Histological classification was based on the immunohistochemical expression of anterior pituitary hormones (FSH, LH, TSH, ACTH, GH, and PRL) in tumor cells. Immunohistochemical (IHC) analyses were performed at the participating centers according to the routine diagnostic protocols. Additional IHC analyses of pituitary-specific transcription factors PIT1 (Novus Cat# NBP1-92,273, RRID: AB_11030310), TPIT (Atlas Antibodies Cat# AMAb91409, RRID: AB_2716678), and SF1 (Abcam Cat# ab217317, RRID: AB_2920891) were performed for cases of non-functioning (NF)-PitNETs that were immunohistochemically negative for all anterior pituitary hormones. For the first surgery specimens, IHC was also performed using monoclonal antibodies against Ki67 (clone MIB1, Agilent Cat# GA626, RRID: AB_2687921) and p53 (clone DO-7, Agilent Cat# GA616, RRID: AB_2889978) at the Department of Clinical Pathology, Uppsala University Hospital, according to the routine diagnostic protocols (p53 not performed in three cases, and Ki67 was not performed in one due to sparse tumor tissue in the FFPE specimens). Two pathologists (JJ and OC-B) independently quantified the Ki67 stainings and the p53 expression with a high concordance. Ki67 was quantified as the percentage of distinctly positive cells among 2,000 tumor cells (focusing on hotspots if present), while p53 was quantified as the total number of distinctly p53 positive cells in 10 HPFs.

Forty six of the fifty tumors from the first surgery cohort could be classified according to the French 5-tiered pituitary tumor classification [45].

### DNA purification

DNA was purified from 10 μm-thick paraffin slides using the GeneRead DNA FFPE Kit (Qiagen, Hilden, Germany) according to the manufacturer’s instructions.

### Methylation profiling, tumor classification, and copy-number variation (CNV) analysis

Methylation data were collected using the Illumina InfiniumMethylation EPIC (850 k) BeadChip Kit (Illumina, San Diego, CA, USA) as previously described [26, 32]. Bisulfite conversion was performed using the Zymo EZ DNA Methylation Kit (Zymo Research, Irvine, CA, U.S.A.), and bisulfite-converted DNA was restored using the Infinium HD FFPE Restore Kit from Illumina. The EPIC BeadChip array was scanned with the iScan from Illumina. IDAT files were uploaded to the MolecularNeuropathology.org server (https://www.molecularneuropathology.org/mnp) for in silico tumor classification and generation of copy-number variation (CNV) plots using workflows v11b4, v12.5, and v12.8, as previously described [6, 7].

CNV profiles calculated from methylation profiling data were obtained using the CNV script files embedded as part of the most recent brain tumor classifier workflow (v12.8). Overall CNV profiles for sample groups were generated by annotating individual CNVs in a cumulative manner, using at least 50% gain or loss for a given chromosomal arm as a cutoff for recording the number of chromosomal arm-level alterations for each sample. CNVs for each specimen were scored as gains, losses, or balances (indicating no events), and converted into a factorized matrix as input for hierarchical clustering of the associated CNV events based on the Jaccard similarity index distance. These distances were calculated using the *ade4* R package [11] and visualized in a heatmap using the *ComplexHeatmap* R package [14, 15].

### Fluorescence in situ hybridization (FISH)

To validate the CNV data derived from the methylation analysis, we performed FISH analysis on 24 specimens, including 21 APT/PC and 3 benign PitNETs, using 2 µm sections from FFPE tissue blocks. Specimens were selected to represent the distinct EPIC-derived copy-number profiles among the APT/PC group. Probe sets XL BCR/ABL1 plus and XL DLEU/LAMP/12cen (MetaSystems Probes, Germany) were used to evaluate the ploidy states of chromosomes 9 and 22, as well as 12 and 13, respectively. Following a melting step at 85 °C for 10 min, probes were hybridized at 37 °C for 20 h using VYSIS HYBrite system. The sections were then diluted in stringency buffer, washed, and dehydrated using ethanol. Analysis was performed with VSViewer (MetaSystems, Germany). For each specimen and probe set, at least ten fields of interest were analyzed.

### Data preprocessing

We analyzed the methylation array data in the R (v 4.3.2) environment using the *ChAMP* R package (v 2.32.0) [28, 44]. First, we imported the raw iDAT files and the sample sheet using the champ.load function. We then set the default quality control parameters and filtered out probes with overlapping SNP positions, sex chromosomes, and detection *p* values > 0.01. Next, we normalized the retained probes using the beta mixture quantile dilation method (BMIQ). For further analysis, we considered only the shared CpGs across the 81 specimens, leading to the selection of 384,629 CpG probes from an initial pool of 865,918 probes. Thereafter, we used the champ.SVD function to assess correlations between the covariates, including slide, array, sex, and age, and the principal components accounting for most of the variation in the data. This analysis revealed correlations between slide, array, and sex and the major principal components. To counteract these batch effects, we used the champ.runCombat function for batch correction. This procedure was also applied to analyze of the subset of 50 first surgery tumor specimens.

### Unsupervised clustering and differential methylation analysis

We used the CpG probes with the top 5000 most variable *β* values (degree of methylation) as input for unsupervised hierarchical clustering. Clustering was based on the complete linkage method, and Pearson correlation was used as the distance method. The clustering and β values were visualized in a heatmap using the Heatmap function embedded in the *ComplexHeatmap* *R* package [14, 15]. Furthermore, we utilized the top 5,000 most variable *β* values as input for three-dimensional unsupervised clustering using principal component analysis (PCA) and t-distributed Stochastic Neighbor Embedding (tSNE), using the *stats*, *rgl,* and *tSNE* R-packages, respectively.

Differential methylation analysis was based on the β values and using the champ. The DMP function considered CpG probes with a nominal *p* value ≤ 1.3 × 10^–7^ (corresponding to genome-wide significance) and an absolute change in *β* value ≥ 0.2 as significantly methylated. Initial analysis of the shared CpGs from 83 specimens (including 65 APT/PC patients and 12 control patients) revealed two specimens as clear outliers. One was the only available specimen from a metastasis in a patient with a silent corticotroph tumor who also had two additional neoplasms: a rare retroperitoneal liposarcoma and colorectal adenocarcinoma, potentially indicating an underlying genetic tumor syndrome. The morphological appearance of this tumor was consistent with a neuroendocrine tumor, and there was distinct TPIT nuclear staining in the tumor cells. This patient was subsequently excluded from the cohort. The other outlier was a paired specimen from a tumor recurrence of a silent PIT1-positive APT, with no other known malignancy or syndrome. The morphological appearance, characterized by lobular architecture and moderate cell atypia, could suggest an immature PIT1-cell lineage tumor. However, the similarity between the first and recurrent specimens argues against dedifferentiation to an immature PIT1-cell lineage tumor over time. The recurrent specimen, which exhibited outlier behavior, was excluded, while the first surgery specimen was further analyzed as part of the first surgery cohort (Fig. 1). For information regarding the methylation class of the two outliers, see the Methylation profiling and tumor classification section in the Results section. The two outlier specimens were not part of the study cohort presented in Table 1.

We compared the degree of methylation between the APT/PC group and the benign tumor group, both in the entire dataset and in the subset of 50 tumor specimens obtained from the first surgery.

### Gene set enrichment analysis of differentially methylated positions between aggressive and metastatic PitNETs

Gene set enrichment analysis (GSEA) was performed using the *msigdbr* and *fgsea* *R* packages. For each comparison of differential methylation, the CpGs were ranked by their β-values, from most positive to most negative. Next, CpGs located outside a gene loci were filtered out. For those CpGs located in multiple positions within the same gene, only the entry with the highest absolute value was kept for further analysis. The final pre-ranked individual genes were then subjected to GSEA. We used collections of human KEGG (Kyoto Encyclopedia of Genes and Genomes, http://www.genome.jp/kegg/) defined gene sets as input for the analysis. GSEA was conducted using 1000 permutations, with the eps set to zero. The minimum and maximum gene set sizes were set to 15 and 500, respectively. Gene sets with FDR ≤ 0.05 were considered as being statistically significantly enriched.

## Results

### Unsupervised hierarchical clustering

#### Unsupervised hierarchical clustering of the entire cohort

**Fig. 2:**
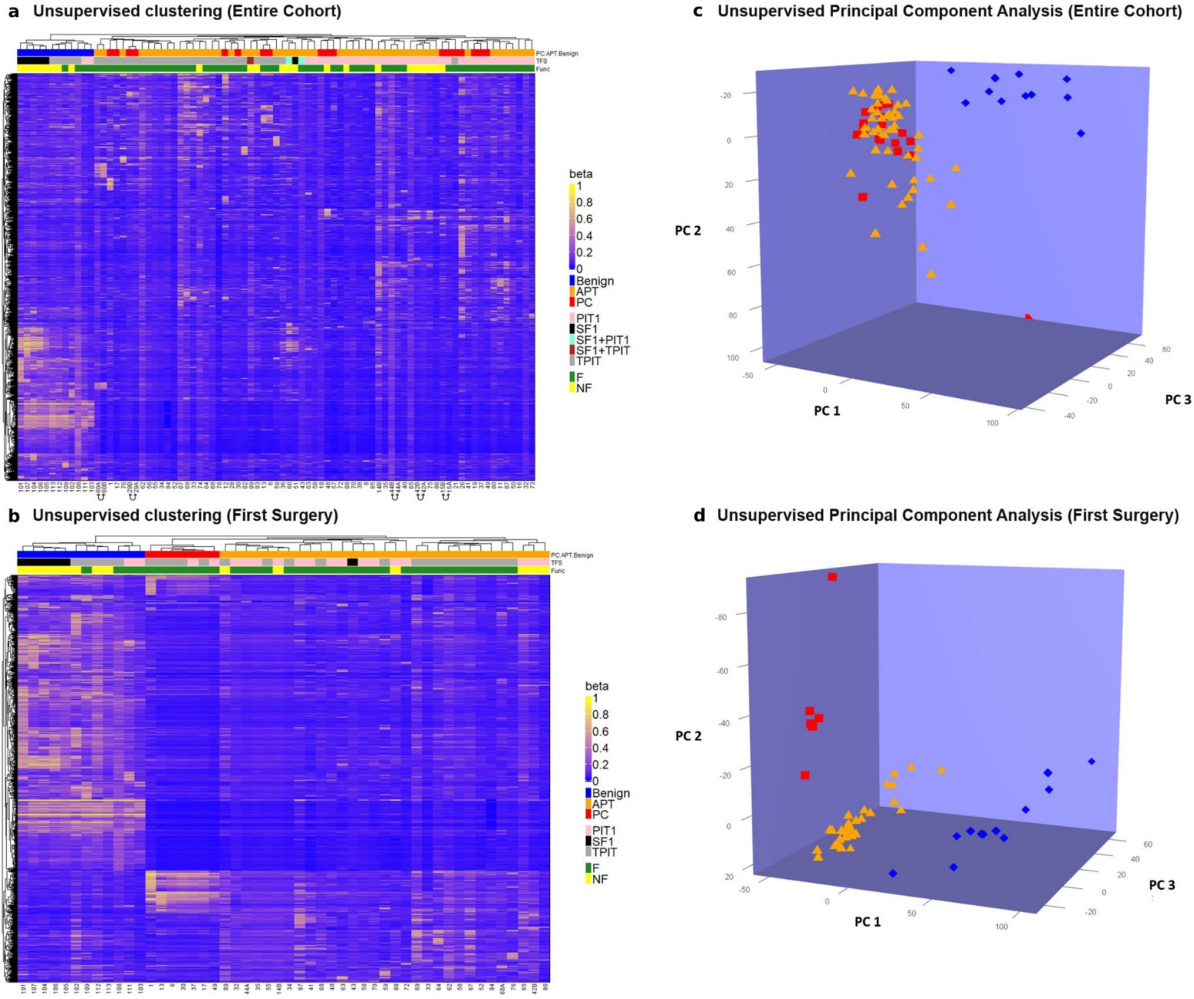
Heatmap showing the degree of methylation (beta value) of the top 5000 most variable CpG sites and the associated hierarchical clustering of specimens from the entire cohort (*n* = 81) (**a**) and the subset of first surgery specimens (*n* = 50) (**b**). Blue and yellow shadings indicate hypomethylation and hypermethylation, respectively. The annotation bar shows the tumor type (benign—blue, APT—orange, and PC—red), transcription factors, and functional status of the tumors. Paired samples are marked with the connecting arrows. Unsupervised principal component analysis (PCA) illustrates clustering of the c) specimens from the entire cohort (*n* = 81) and d) the subset of first surgery specimens (*n* = 50) based on the CpG sites with the top 5,000 variable β values. The blue diamonds, orange triangles, and red boxes represent the benign, APT, and PC specimens, respectively. PC1, PC2, and PC3 are the axes of the 1st, 2nd, and 3rd principal components (PCs), respectively. In the PCA of the entire cohort, the first three PCs explain 20.2%, 9.2%, and 4.8% of the total variance, while in the PCA of the first surgery specimens, the 1st, 2nd, and 3rd PCs explained 38.6%, 8.4% and 6.8% of the variance, respectively

In the entire cohort of 81 specimens (Fig. 1), unsupervised hierarchical clustering based on the top 5000 most variable CpG sites revealed complete separation of the APT/PC group from the benign tumor group (Fig. 2a). There was no distinction between APT and PC. The APT/PC group could be further separated into three pituitary cell lineage-driven subclusters: (1) the PIT1 subcluster, which contained 35 PIT1 specimens (26 APT, out of which 7 NF-APT, and 9 PC, out of which two NF-PC) and one F-TPIT PC; (2) the TPIT subcluster, which contained 29 TPIT specimens (21 APT, out of which three silent and 8 F-PC) and one sample from the TPIT/SF1-positive NF-APT, also classified as ACTH-producing adenoma in the Heidelberg classifier; and (3) the SF1 subcluster, which included one SF1 NF-APT sample and two SF1/PIT1 APT specimens (one NF-APT and one F-APT). Within the benign group, clear separation into SF1, TPIT, and PIT1 tumors was noted. The unsupervised clustering in the APT/PC group did not reveal any separation between F- and NF-PitNETs, whereas benign tumors tended to group into F- and NF-PitNET.

Paired specimens from five patients (3 APTs and 2 PC with intervals between surgeries of 9–16 and 4–11 years, respectively) had very similar methylation profiles and were positioned next to each other in the methylation heatmap (Fig. 2a).

### Unsupervised hierarchical clustering of the first surgery specimens

To explore the potential use of methylation analysis as an early predictor of tumor aggressiveness, a subcohort containing 50 specimens from the first surgery (38 APT/PC and 12 benign tumors) was analyzed. This approach revealed a complete separation of the APT/PC group from the benign tumors as well as a clear separation of the PC within the APT/PC group (Fig. 2b).

Unsupervised clustering showed separation with respect to pituitary cell lineages (TFs) in the benign tumor group, but not in the APT/PC group (Fig. 2b).

### Principal component analysis

To further study the methylome-based clustering, we performed a principal component analysis (PCA) to verify the relationships among benign tumors, APT, and PC in both the whole cohort and the first surgery cohort, as visualized by hierarchical clustering. PCA revealed clustering of aggressive and metastatic tumors separated from benign tumors in the entire cohort (Fig. 2c). In the cohort of the first surgery specimens, three clusters corresponding to APT, PC, and benign tumors were defined (Fig. 2d). The methylome-based clustering was also presented using t-SNE (Supplementary Fig. 1a and 1b).

## Differential methylation analysis

### Differential methylation analysis of the entire cohort

**Fig. 3:**
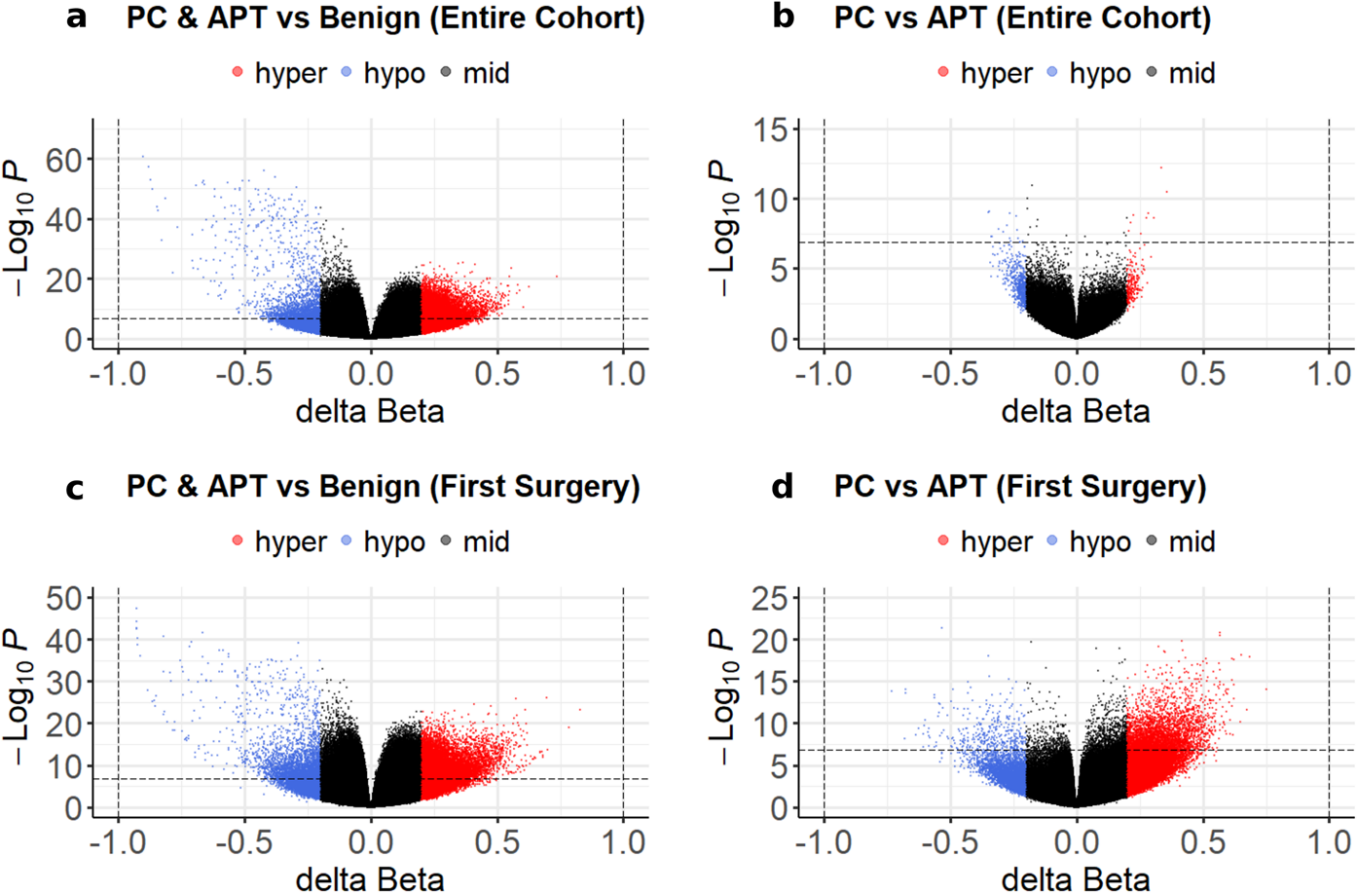
Volcano plots showing differential methylation analysis results in APT/PCs vs. benign PitNETs. The X-axis shows the ∆β-value (difference in beta value), and the Y-axis shows the associated –log_10_ *p* values. CpGs with ∆β ≤ − 0.2 or ∆β ≥ 0.2 are shown in blue (hypomethylated), red (hypermethylated), and gray (not deviating from the threshold), respectively. The dotted line shows the global significance threshold (*p* = 1.3 × 10^–7^). **a** All specimens comparing APT/PC (*n* = 69) vs. benign tumors (*n* = 12); **b** all specimens comparing PC (*n* = 18) vs APT (*n* = 51); **c** the first surgery specimens comparing APT/PC (*n* = 38) vs. benign tumors (*n* = 12); and **d** the first surgery specimens comparing PC (*n* = 7) vs. APT (*n* = 31)

To identify the features of DNA methylation associated with aggressive behavior, we analyzed differentially methylated positions (DMPs) between the aggressive (APT/PC) and benign groups. A total of 9066 significant DMPs were detected with a *p* value < 1.3 × 10^–7^ and an absolute change in *β* value ≥ 0.2, when APT/PC and benign tumors were compared. Among APT/PCs, 7394 DMPs exhibited hypermethylation, whereas 1672 DMPs displayed hypomethylation (Fig. 3a).

A comparison of DNA methylation between the PC and APT revealed 24 significantly DMPs; 9 DMPs displayed hypermethylation, whereas 15 DMPs exhibited hypomethylation in the PC (Fig. 3b).

### Differential methylation analysis of the first surgery specimens

A total of 12,235 significant DMPs were detected with a *p* value < 1.3 × 10^–7^ and an absolute change in *β* value ≥ 0.2, when the APT/PC specimens from the first surgery and benign tumors were compared. Among these, 9628 DMPs exhibited significant hypermethylation, whereas 2607 DMPs displayed hypomethylation in APT/PC (Fig. 3c).

A comparison between first surgery PC and APT revealed 3402 significantly DMPs, 3046 of which were significantly hypermethylated and 356 of which were hypomethylated DMPs in the first surgery PC specimens (Fig. 3d).

### Gene set enrichment analysis of the differentially methylated positions between aggressive and metastatic PitNETs

To further explore the differences between APT and PC, we analyzed significantly enriched gene pathways associated with the DMPs that differed between the two groups. In the specimens from the first surgery, there were 33 significantly positive enriched pathways, compared to 14 in the entire cohort. The ten most relevant pathways are listed in Table 2, with several pathways being common to both the first surgery and the entire cohort. Additional information on the entire gene sets associated with the DMPs that differed between APT and PC can be found in the Supplementary Tables 1 and 2.

**Table 2:** Relevant significantly enriched gene sets associated with the DMPs that differed between aggressive and metastatic PitNETs in the first surgery specimens (upper part of the table) and in the entire cohort (lower part of the table)

Hypermethylated and positive enriched in PC	pval	padj	NES	size
KEGG Cell adhesion molecules CAMS	6.26E-07	5.63E-05	1.65	122
KEGG Axon guidance	6.51E-07	5.63E-05	1.65	122
KEGG Pathways in cancer	1.43E-06	8.24E-05	1.44	313
KEGG Neuroactive ligand receptor interaction	6.39E-06	0.00028	1.44	249
KEGG Focal adhesion	7.16E-05	0.0015	1.44	191
KEGG Adherens junction	8.97E-05	0.0017	1.64	67
KEGG Calcium signaling pathway	0.00025	0.0039	1.40	166
KEGG Leukocyte transendothelial migration	0.0019	0.019	1.43	108
KEGG ECM receptor interaction	0.0019	0.019	1.49	83
KEGG Gap junction	0.0063	0.036	1.40	83

Hypermethylated and positive enriched in PC	pval	padj	NES	size
KEGG Axon guidance	1.60E-06	0.00028	1.53	122
KEGG Calcium signaling pathway	3.22E-06	0.00028	1.44	166
KEGG Neuroactive ligand receptor interaction	0.00010	0.0045	1.31	249
KEGG Regulation of actin cytoskeleton	0.00013	0.0045	1.34	197
KEGG MAPK signaling pathway	0.00030	0.0066	1.29	251
KEGG Cell adhesion molecules cams	0.0018	0.035	1.33	122
KEGG ECM receptor interaction	0.0034	0.049	1.36	83
KEGG Wnt signaling pathway	0.0052	0.064	1.27	145
KEGG Hedgehog signaling pathway	0.0057	0.064	1.40	55
KEGG Leukocyte transendothelial migration	0.011	0.10	1.28	108

## Copy-number variation analysis

CNV events were considered frequent if detected in more than 50% of the specimens. Overall, CNV patterns in APT/PCs were dominated by CNV events affecting whole-chromosome arms (arm-length gains and losses) or entire chromosomes, while focal CNV events, such as gene amplifications and gene losses, were rare. In contrast, none or very few CNV events were present in benign tumors.

To evaluate ploidy states of chromosomes 9, 12, 13, and 22 in tumor cells, FISH analysis was performed on 24 specimens selected to represent the distinct EPIC-derived CNV profiles, which were highly enriched for whole-chromosomal gains among the APT/PC. Additionally, we inferred the copy numbers of the remaining chromosomes from the EPIC-derived CNV profiles. In summary, tetraploidy, often involving chromosomes 5, 7, 9, 12, 14, and 20, emerged as the overall CNV landmark of APT/PC with respect to copy numbers (Supplementary Table 3).

### Differences in the cumulative CNV profile between APT/PCs and benign tumors

**Fig. 4:**
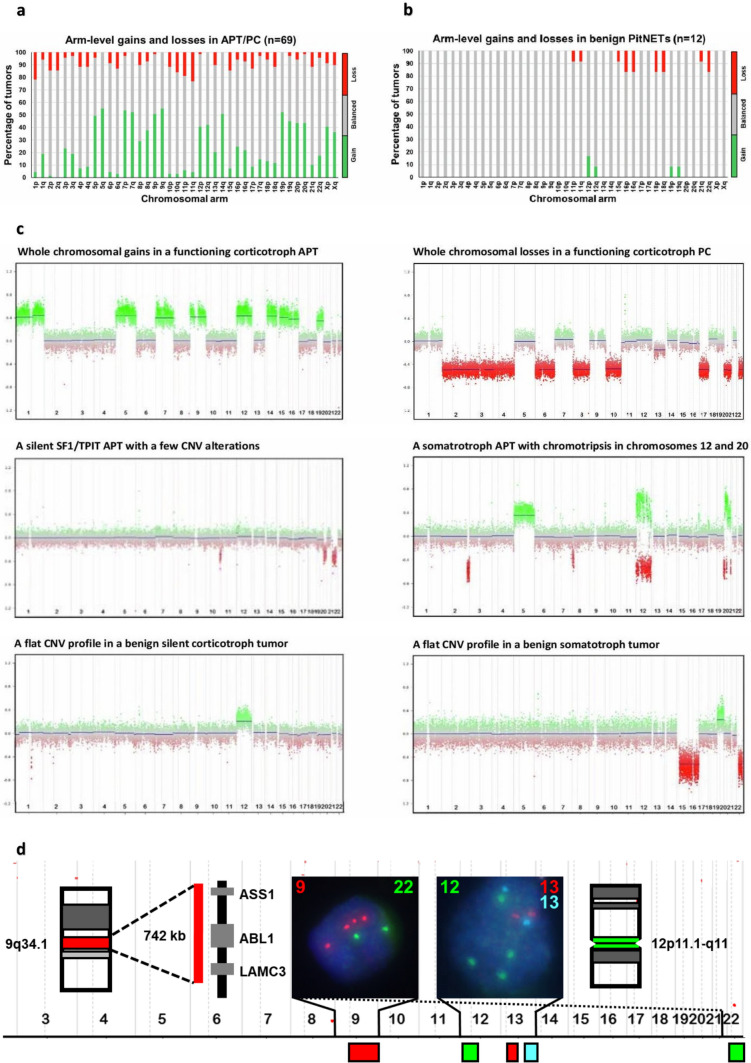
Cumulative CNV profiles showing abundant chromosomal alterations with a number of arm-level gains (green) and losses (red) in APT/PCs (**a**) in comparison to benign PitNETs, which were dominated by balanced chromosomes (gray) (**b**). The distribution of CNV alterations on chromosomal arms is shown on the X-axis, while the percentage of tumors with CNV alterations is shown on the Y-axis. **c** Examples showing a variation of distinct CNV profiles between APT/PCs and benign PitNETs. Chromosomes with a normal copy number (*n* = 2) exhibit an even distribution of data points around the calculated baseline, while gains (*n* > 2) and losses (*n* < 2) are depicted in green and red, respectively. Full vertical lines separate individual chromosomes, while stippled lines indicate the separation of the p and q arms. Chromosome numbers are shown on the X-axis of each CNV. d) Diagram showing the positions of FISH probes targeting chromosomes 9, 12, 13, and 22 with detailed information on probe locations for 9q arm and 12 centromere. Left FISH picture insert shows tumor cell with tetraploidy of chromosome 9 in red and normal ploidy of chromosome 22 in green, while right FISH picture insert shows tetraploidy of chromosome 12 in green and normal ploidy of chromosome 13 in red/cyan, respectively

Striking differences in CNV patterns were demonstrated when comparing APT/PC to benign tumors. Arm-level alterations were much more frequent and affected more chromosomes in APT/PC [median 16, range 0–41] than in benign tumors [median 1, range 0–6]. Generally, the CNV events in APT/PC group affected chromosomal arms and whole chromosomes, with gains occurring more frequently than losses (Fig. 4a). At least 40% of specimens showed gains involving chromosomes 5, 7, 9, 12, 19, and 20 as the strongest overall trend. Notably, almost identical CNV patterns were detected in the whole APT/PC cohort and in the 50 first surgery specimens. In contrast to APT/PC, CNV profiles from benign PitNETs were dominated by normal chromosomal copy numbers with only a few alterations, which were detected in less than 20% of cases (Fig. 4b).

Although APT/PC showed highly disrupted CNV profiles in general, pronounced individual variations were detected (Fig. 4c). Chromothripsis was detected in five PIT1 tumors (three lactotroph and two somatotroph tumors; with two being PC and three APT). Besides a few nonrecurrent chromothripsis events, this phenomenon repeatedly involved chromosome 11 in all three lactotroph tumors and chromosome 12 in both somatotroph tumors. Examples of different individual CNV profiles are shown in Fig. 4c. Tetraploidy of chromosome 9 and 12 in tumor cells from two representative specimens by FISH analysis is illustrated in Fig. 4d.

### Differences in CNV profiles related to tumor type and functional status

**Fig. 5:**
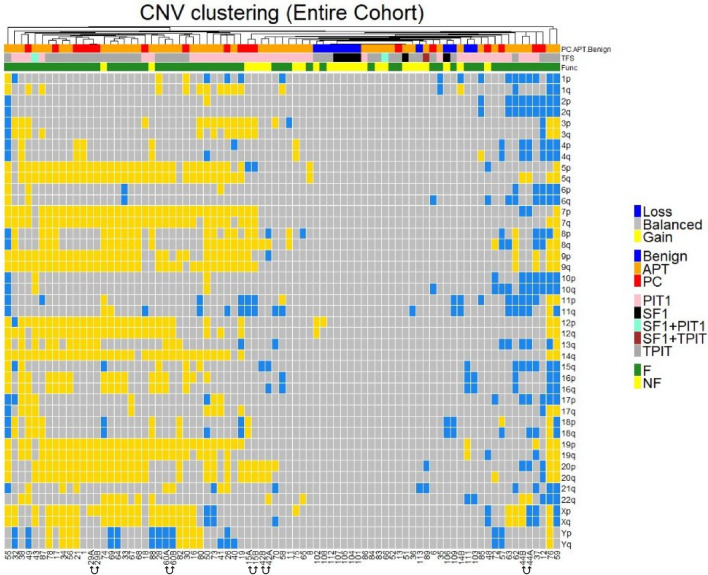
Heatmap showing unsupervised CNV clustering in specimens from the entire cohort. Losses, gains, and balanced CNVs are shown in blue, yellow, and gray, respectively. Chromosome regions are ordered by number and displayed in rows, while the specimens are organized by hierarchical clustering and presented in columns. The annotations of the specimens with respect to tumor group (APT/PC/benign), transcription factor, and functional status are shown in the bars above the heatmap. Paired samples are marked with connecting arrows

The APT/PC of different cell lineages and functional statuses (hormone-secreting and nonsecreting) showed distinct CNV patterns (Fig. 5).

Cushing APT/PC specimens in general showed very disrupted CNV profiles, dominated by frequent gains of chromosomes 5, 7, 9, 12, 14q, 19, 20, and X, although variations were observed. The cumulative CNV pattern in acromegaly APT/PCs was dominated by frequent gains of chromosomes 3p, 7, and 20p, along with frequent losses of chromosome 11q. For lactotroph tumors, frequent gains of chromosomes 3p, 5, 7, 8q, 9, 14q, and 19p were detected. Notably, two pure TSH-secreting APT/PC exhibited more frequent losses (chromosomes 1p, 2, 6q, 8q, 10q, 11, and 13q) than gains (1q, 11p, and X). A single gonadotroph tumor in our APT/PC cohort had a normal chromosomal copy number. NF-tumors demonstrated quiet CNV profiles without frequent large-scale gains or losses in both APT/PC and benign tumors. Nonetheless, compared to benign tumors, NF-APT/PCs showed more CNV events. Benign NF-PitNETs showed only a few rare CNV events, while 63% (5/8) of the tumors demonstrated no alterations. The paired samples from five patients with APT/PC had almost identical CNV profiles and were positioned next to each other in the CNV heatmap (Fig. 5).

### Correlation between methylation and CNV profiles and histological tumor features in the first surgery specimens

A significant number of the first surgery APT/PC specimens showed only slight-to-moderate cell atypia (25/38), < 3 mitoses per 10 HPF (17/38), relatively low Ki67 (< 3% in 9 and < 10% in 23 of 37 cases), and p53 expressed in < 10 cells/10 HPF (23/35). Among 34 APT/PC that could be classified according to the French classification, 15 were classified as grade 2b, 14 as grade 2a, four tumors were classified as grade 1b and one as grade 1a. The relationships between methylation data, histological data, and the French 5-tiered classification are presented in Supplementary Fig. 2.

Tumors with quiet CNV profiles mainly showed mild-to-moderate cell atypia, low mitotic count, and lower Ki67 indices. However, a few exceptions were noted, most often in functioning corticotroph tumors. In contrast, APT/PC with disturbed CNV profiles exhibited pronounced atypia and moderate-to-high mitotic indices. Tumors characterized by gains in CNV profiles were more likely to present higher Ki67 indices and p53 expression in > 10 cells/10 HPF compared to tumors dominated by losses. (Supplementary Fig. 3).

### Methylation profiling and tumor classification

According to the Heidelberg methylation classifier [7], 70% (50/71) of all APT/PC specimens matched the pituitary adenoma class, and 63% (45/71) of the specimens even attained subclassification that reflected current histological classification based on immunohistochemical expression of pituitary hormones and TFs when a classification threshold of 0.84 was applied [6, 7]. Notably, corticotroph tumors achieved the highest match of 90% (26/29 specimens). In 20 APT/PC specimens, the methylation subtypes corresponded to the correct immunohistochemical tumor type, or at least, to the correct cell lineage; however, their calibration scores were < 0.84. In four of the five patients with paired samples, at least one of the paired samples had a score > 0.84 corresponding to the correct tumor type. In the remaining case with paired samples, a patient with a PC, one specimen was not classified as a PitNET, while the other indicated a somatrotroph tumor (score 0.7), corresponding to the clinical and immunohistochemical profile of the tumor. The two specimens had similar methylation and CNV profiles and were positioned next to each other in the methylation and CNV heatmap. Among the benign PitNETs, 92% (11/12) matched the pituitary adenoma class and subclass.

Of the two outliers, one was a metastasis, and the only specimen available from a patient with a non-functioning corticotroph tumor. The methylation profile matched the methylation class “pituitary adenoma”; however, a match score to the tumor subtype was not achieved, although the highest score was 0.77 for the corticotroph subtype. The other outlier specimen, a recurrent tumor from a patient with a silent PIT1-positive APT, tended to match the “pituitary adenoma” class (score 0.5), TSH-subtype (score 0.3). In contrast, the first specimen from the same patient, which did not behave as an outlier, matched the TSH-producing adenoma with a score of 0.92.

## Discussion

In this study, we performed genome-wide methylation profiling in the largest international cohort of aggressive and metastatic pituitary neuroendocrine tumors reported to date. A clear separation of APT/PC from benign tumors was evident already in the first surgical specimens and distinct CNV patterns were associated with tumor aggressiveness. Thus, DNA methylation analysis could be a valuable tool for the early identification of patients at risk of developing aggressive and metastatic PitNETs.

Unsupervised hierarchical clustering, principal component analysis, and t-SNE revealed a complete separation of the APT/PC group from benign tumors when applied to the entire cohort. The majority of differentially methylated positions (DMPs) were hypermethylated, suggesting that DNA hypermethylation is the main epigenetic phenomenon involved in the development of an aggressive phenotype. Methylation signatures classify PitNETs according to cell lineages [29, 30]. To the best of our knowledge, genome-wide methylation data on a large cohort of aggressive and metastatic pituitary tumors have not been reported. Previous studies covered only candidate genes [16] or were performed on tumors showing invasiveness [20, 24, 29, 30, 39], postsurgical recurrence [39], or postsurgical growth requiring reintervention [18]. However, tumors refractory to all standard therapies and metastatic PitNETs were usually not included in these studies. In the study by Neou et al. [30], samples from a few tumors categorized as APT clustered based on cell lineage, but not according to aggressive behavior by use of unsupervised hierarchical analysis of the top 2,500 most variable CpG features. An explanation for the different outcomes may be the large number of aggressive (48) and metastatic (16) PitNETs in our study, as well as the strict selection of patients according to the ESE guidelines [33].

Interestingly, when only specimens from the first surgery were analyzed, distinct APT and PC methylation clusters were observed. The number of DMPs that differed between aggressive and metastatic PitNETs was considerably higher in the first surgery specimens (3,402 DMPs) compared to the entire cohort (24 DMPs). One potential explanation for this finding could be that a subset of the APT acquired additional aberrations over time, obscuring the distinct patterns present earlier in tumor development. However, this speculation is not supported by our cohort, as paired specimens from five patients (two PC patients with specimens from pituitary surgery and from metastasis, and three APT patients with specimens from different pituitary surgeries with at least 10 years between the surgeries) were positioned next to each other within the methylome heatmap. Among these five patients, one patient with metastatic tumor received radiotherapy before both surgeries, while the other four patients received radiotherapy between the two surgeries. Based on the clustering results, radiotherapy did not significantly interfere with the DNA methylation profile. In addition, when examining the significantly enriched pathways associated with the DMPs that differed between APTs and PCs, it seems that aggressive and metastatic PitNETs share oncogenic mechanisms during tumor progression. These pathways included genes that regulate tumor progression and metastasis, e.g., genes related to cell adhesion, TGF-beta, Wnt, Hedgehog, MAPK and RTK signaling pathways, among others, and were similar or overlapping between the first surgery cohort and the entire cohort. Thus, we have no good explanation for distinct clustering of APT and PC observed in the first surgery cohort. Analysis of a larger number of treatment-naïve metastatic PitNETs and comparisons between epigenetic and genetic alterations could provide more insights into this observation.

None of the patients with metastatic PitNETs had known metastasis at the time of the first surgery. The distinct clustering of the PC specimens from the first surgery suggests that methylation profiling may predict the metastatic potential of a PitNET at an early stage. In a clinical setting, such a finding may be an indication for intensified follow-up and screening for metastases, as well as earlier initiation of adjuvant radio- and/or chemotherapy.

DNA methylation-derived CNV profiles typically showed disrupted CNV signatures, including arm-length and whole-chromosomal events, with a predominance of whole-chromosomal gains as the most recurrent CNV pattern in our APT/PC cohort. However, a small subset of functioning and non-functioning APT/PC, mostly of the corticotroph type, along with a few PIT1 tumors and a single gonadotroph tumor, demonstrated flat CNV profiles, similar to the majority of benign tumors. An association between extensive CNVs and tumor size and invasiveness in corticotroph tumors has been reported [43, 46], but not in relation to persistent/recurrent disease [46]. A single PC in one study showed disrupted CNV, while no association between CNV pattern and recurrence status in benign tumors of different types was found [2]. Likewise, others did not find any association between CNVs and parameters related to aggressiveness [17, 30, 41], possibly due to differences in patient selection and the low number of truly aggressive tumors. Recently, a study based on next-generation sequencing demonstrated widespread chromosomal loss of heterozygosity in 26 treatment-refractory PitNETs, mostly of the corticotroph type [23]. The finding of almost identical CNV profiles between paired specimens obtained from the same patient at different time points in the present study suggests that CNV alterations occur early, remain constant during the disease progression and do not seem to be affected by radiotherapy. Thus, we propose that a disrupted CNV profile with numerous arm or whole-chromosome alterations in a pituitary tumor provides prognostic information and should raise suspicion of APT/PC.

Although it was not the main focus of our study, we also analyzed the relationship between the CNV patterns, genome-wide methylation signatures, and the histological and functional types of APT/PC. The corticotroph tumors in our cohort could be subdivided into a highly CNV-disrupted APT/PC-dominated subgroup and a smaller CNV-deficient subgroup containing mostly benign tumors, as well as a few TPIT-positive APT/PCs, both silent and functioning. Our data are similar to previously described variations in CNV patterns in corticotroph tumors [1, 46]. CNV events were less frequent in somatotroph tumors than in corticotroph tumors, which is consistent with data from another cohort [21]. A few corticotroph APT/PCs as well as a single gonadotroph APT and a plurihormonal SF1/PIT1-positive APT showed normal chromosome copy numbers. This phenomenon was also observed in benign PitNETs, both in our cohort and in other studies [3, 30]. Overall, there was an association between fewer or absent CNV events and the non-functioning status of PitNETs in our cohort, similar to findings in the previous reports [3, 17, 21, 30, 36, 47].

Our assessment of ploidy states in the tumor cells, which combined EPIC-derived CNV profiles with FISH analysis of 24 selected specimens to establish exact copy numbers, revealed whole-chromosomal tetraploidy often involving chromosomes 3, 5, 7, 8, 9, 12, 14, 19, and 20 in APT/PC, as has been shown previously [30, 46].

Another CNV phenomenon observed in our cohort was chromothripsis, which is characterized by many clustered chromosomal rearrangements focused on localized genomic regions resulting in oscillations between “gain and loss” states [42]. In line with the other reports [17, 37, 47], we observed this phenomenon only in PIT1 tumors (5 out of a total of 32, 3 APT, 2 PC), with chromosome 11 being affected in the three lactotroph tumors, and chromosome 12 in both somatotroph tumors. The specimens were from the first surgery without prior radiotherapy, which argues against a previous speculation that chromothripsis is induced by irradiation [27]. Further studies are warranted to clarify the implications and importance of this phenomenon.

Unsupervised hierarchical clustering revealed that APT/PC clustered according to the pituitary transcription factors but separated from benign tumors of the same adenohypophyseal cell lineage. This finding confirms that the methylation fingerprints of the cell of origin are preserved throughout the neoplastic transformation and disease progression, as speculated before [7]. The APT/PCs mainly followed the classification pattern for PitNETs, with more than 70% of all APT/PC specimens matching the “pituitary adenoma” class according to the Heidelberg methylation classifier [7] when a calibration score of 0.84 was applied. Most specimens attained a subclassification that reflected the current cell lineage-based WHO classification [48]. Approximately 30% of tumors in our APT/PC cohort were also classified as PitNETs and followed the immunohistochemical tumor type; however, their calibration score was < 0.84. The lower calibration score in this subset of APT/PC is likely related to the profound epigenetic alterations often associated with aggressive tumor behavior. Conversely, 92% of benign PitNETs matched the “pituitary adenoma” class.

Silent corticotroph tumors are regarded as potentially more aggressive type of PitNET in the current WHO classification [48]. A previous methylation study revealed a separation between silent and functioning corticotroph tumors, with the presence of a third cluster representing tumors causing subclinical Cushing disease [34]. The six silent corticotroph tumors in our cohort (3 APT, 3 benign tumors) were scattered among the functioning TPIT tumors. Five of these tumors showed few or no CNV alterations, while one APT had many gains, similar to the functioning corticotroph APT/PC. The clustering of three benign silent TPIT tumors among the benign PitNETs, and three aggressive silent TPIT tumors within the APT/PC group suggests that aggressive silent corticotroph tumors might be identified by their epigenetic profiles. However, given the small number of cases, more data are needed to support this observation.

Finally, to explore whether methylation profile and CNV patterns may potentially be informative as prognostic markers in histologically benign-looking PitNETs, we correlated methylation and CNV data with tumor grade as defined by the French 5-tiered classification [45]. Of the 34 first surgery APT/PC specimens, 15 (44%) were classified as grade 2b (high-risk proliferative and invasive tumors), while the majority exhibited only slight-to-moderate cell atypi and no signs of increased cell proliferation or p53 expression. This indicates that methylation analysis may help identify aggressive and metastatic PitNETs that present with benign histology and without increased proliferation at the time of the first surgery.

### Limitations of the study

Specimens from lactotroph tumors were not included in our benign cohort, since these tumors rarely undergo surgery unless they are dopamine agonist resistant, and thus do not fulfill the strict criteria for benign tumors in the present study. We have not performed gene analyses that would enable exploration of the association between genetic and epigenetic events in more detail. Integration of the genetic data and validation in larger cohorts covering the entire spectrum of clinical behaviors of PitNETs would increase statistical power and enable analysis of confounding factors.

## Conclusion

Genome-wide methylation analysis revealed a clear separation and different copy-number variation profiles between aggressive/metastatic and benign pituitary neuroendocrine tumors. This finding provides a potential tool for early identification and, consequently, optimized follow-up and timely introduction of radio- and pharmacological therapy for patients at risk of aggressive or metastatic pituitary tumors. Epigenetic data, including CNV analysis, may enable the development of an integrated multiparameter prognostic classification of PitNETs, similar to the current classification of central nervous system tumors.

## Supplementary Information

Below is the link to the electronic supplementary material.

Supplementary file1 (TIFF 5772 KB)

Supplementary file2 (TIFF 3667 KB)

Supplementary file3 (TIFF 2377 KB)

Supplementary file4 (XLSX 59 KB)

Supplementary file5 (XLSX 60 KB)

Supplementary file6 (XLSX 12 KB)

## Data Availability

The datasets used and/or analyzed during the current study are available from the corresponding author on reasonable request.
