# Correction: Genome‑wide methylation profiling differentiates benign from aggressive and metastatic pituitary neuroendocrine tumors

**DOI:** 10.1007/s00401-024-02838-3

**Published:** 2025-01-02

**Authors:** Jelena Jotanovic, Henning Bünsow Boldt, Mark Burton, Marianne Skovsager Andersen, Daniel Bengtsson, Thomas Olsson Bontell, Bertil Ekman, Britt Edén Engström, Ulla Feldt-Rasmussen, Ansgar Heck, Antonia Jakovcevic, Jens Otto L. Jørgensen, Ivana Kraljevic, Jacek Kunicki, John R. Lindsay, Marco Losa, Paul Benjamin Loughrey, Dominique Maiter, Maria Maksymowicz, Emilija Manojlovic-Gacic, Jens Pahnke, Stephan Petersenn, Maria Petersson, Vera Popovic, Oskar Ragnarsson, Åse Krogh Rasmussen, Zita Reisz, Wolfgang Saeger, Camilla Schalin-Jäntti, David Scheie, Maria Rosa Terreni, Olli Tynninen, Ben Whitelaw, Pia Burman, Olivera Casar-Borota

**Affiliations:** 1 Department of Immunology, Genetics and Pathology, Uppsala University, Uppsala, Sweden; 2 Department of Clinical Pathology, Uppsala University Hospital, Uppsala, Sweden; 3 Department of Clinical Research, University of Southern Denmark, Odense, Denmark; 4 Department of Pathology, Odense University Hospital, Odense, Denmark; 5 Department of Clinical Research, Faculty of Health Sciences, University of Southern Denmark, Odense, Denmark; 6 Department of Clinical Genetics, Odense University Hospital, Odense, Denmark; 7 Clinical Genome Center, University of Southern Denmark, Odense, Denmark; 8 Department of Endocrinology, Odense University Hospital, Odense, Denmark; 9 Department of Health, Medicine and Caring Sciences, Linköping University, Linköping, Sweden; 10 Department of Internal Medicine, Kalmar County Hospital, Kalmar, Sweden; 11 Department of Physiology, Institute of Neuroscience and Physiology, Sahlgrenska Academy, University of Gothenburg, Gothenburg, Sweden; 12 Department of Clinical Pathology, Sahlgrenska University Hospital, Gothenburg, Sweden; 13 Department of Endocrinology in Linköping, Department of Internal Medicine in Norrköping, and Department of Health, Medicine and Caring Sciences, Linköping University, Linköping, Sweden; 14 Department of Medical Sciences, Endocrinology and Mineral Metabolism, Uppsala University, Uppsala, Sweden; 15 Department of Endocrinology and Diabetes, Uppsala University Hospital, Uppsala, Sweden; 16 Department of Medical Endocrinology and Metabolism, Rigshospitalet, Copenhagen, Denmark; 17 Institute of Clinical Medicine, Faculty of Health Research Sciences, Copenhagen University, Copenhagen, Denmark; 18 Section for Specialized Endocrinology, Oslo University Hospital, Oslo, Norway; 19 Department of Pathology and Cytology, University Hospital Center Zagreb, Zagreb, Croatia; 20 School of Medicine, University of Zagreb, Zagreb, Croatia; 21 Department of Endocrinology and Internal Medicine, Aarhus University Hospital, Aarhus, Denmark; 22 Department of Endocrinology, University Hospital Center Zagreb, Zagreb, Croatia; 23 Department of Neurosurgery, Maria Sklodowska-Curie National Research Institute of Oncology, Warsaw, Poland; 24 Regional Centre for Endocrinology and Diabetes, Royal Victoria Hospital, Belfast Health and Social Care Trust, Belfast, UK; 25 Department of Neurosurgery, IRCCS San Raffaele Scientific Institute, Vita-Salute University, Milan, Italy; 26 Patrick G Johnston Centre for Cancer Research, Queen’s University Belfast, Belfast, UK; 27 Department of Endocrinology and Nutrition, UCL, Cliniques Universitaires Saint-Luc, Brussels, Belgium; 28 Department of Cancer Pathomorphology, Maria Sklodowska-Curie National Research Institute of Oncology, Warsaw, Poland; 29 Institute of Pathology, Faculty of Medicine, University of Belgrade, Belgrade, Serbia; 30 Translational Neurodegeneration Research and Neuropathology Lab, Department of Clinical Medicine, Medical Faculty, University of Oslo, Oslo, Norway; 31 Section of Neuropathology Research, Department of Pathology, Clinics for Laboratory Medicine, Oslo University Hospital, Oslo, Norway; 32 ENDOC Center for Endocrine Tumors, Hamburg, Germany; 33 University of Duisburg-Essen, Essen, Germany; 34 Department of Endocrinology, Karolinska University Hospital, Stockholm, Sweden; 35 Department of Molecular Medicine and Surgery, Karolinska Institutet, Stockholm, Sweden; 36 Medical Faculty, University of Belgrade, Belgrade, Serbia; 37 Department of Endocrinology, Sahlgrenska University Hospital, Gothenburg, Sweden; 38 Department of Internal Medicine and Clinical Nutrition, Institute of Medicine, Sahlgrenska Academy, University of Gothenburg, Gothenburg, Sweden; 39 Wallenberg Centre for Molecular and Translational Medicine, Institute of Medicine, University of Gothenburg, Gothenburg, Sweden; 40 Department of Nephrology and Endocrinology, Rigshospitalet, Copenhagen University Hospital, Copenhagen, Denmark; 41 Department of Clinical Neuropathology, King’s College Hospital, NHS Foundation Trust, London, UK; 42 Institute of Neuropathology, University Medical Center Hamburg-Eppendorf, Hamburg, Germany; 43 Institute of Pathology, University Medical Center Hamburg-Eppendorf, Hamburg, Germany; 44 Endocrinology, Abdominal Center, Helsinki University Hospital, Helsinki, Finland; 45 University of Helsinki, ENDO-ERN (European Reference Network On Rare Endocrine Conditions), Helsinki, Finland; 46 Department of Pathology, Rigshospitalet, Copenhagen University Hospital, Copenhagen, Denmark; 47 Department of Clinical Medicine, University of Copenhagen, Copenhagen, Denmark; 48 Department of Pathology, IRCCS San Raffaele Scientific Institute, Vita-Salute University, Milan, Italy; 49 Department of Pathology, Helsinki University Hospital, University of Helsinki, Helsinki, Finland; 50 Department of Endocrinology, King’s College Hospital, NHS Foundation Trust, London, UK; 51 Department of Endocrinology, Skåne University Hospital, Lund University, Malmö, Sweden

**Correction: Acta Neuropathologica (2024) 148:68** 10.1007/s00401-024-02836-5

In the original publication of this article, upper and lower part of Table 2 was incorrectly formatted. The incorrect and correct version of Table 2 are shown in this correction article. The original article has been corrected.

**Incorrect Table 2.**

**Table 2:** Relevant significantly enriched gene sets associated with the DMPs that differed between aggressive and metastatic PitNETs in the first surgery specimens (upper table) and in the entire cohort (lower table)

Hypermethylated and positive enriched in PC	pval	padj	NES	size
KEGG cell adhesion molecules CAMS	6.26E-07	5.63E-05	1.65	122
KEGG axon guidance	6.51E-07	5.63E-05	1.65	122
KEGG pathways in cancer	1.43E-06	8.24E-05	1.44	313
KEGG neuroactive ligand receptor interaction	6.39E-06	0.00028	1.44	249
KEGG focal adhesion	7.16E-05	0.0015	1.44	191
KEGG adherens junction	8.97E-05	0.0017	1.64	67
KEGG calcium signaling pathway	0.00025	0.0039	1.40	166
KEGG leukocyte transendothelial migration	0.0019	0.019	1.43	108
KEGG ECM receptor interaction	0.0019	0.019	1.49	83
KEGG gap junction	0.0063	0.036	1.40	83
KEGG axon guidance	1.60E-06	0.00028	1.53	122
KEGG calcium signaling pathway	3.22E-06	0.00028	1.44	166
KEGG neuroactive ligand receptor interaction	0.00010	0.0045	1.31	249
KEGG regulation of actin cytoskeleton	0.00013	0.0045	1.34	197
KEGG MAPK signaling pathway	0.00030	0.0066	1.29	251
KEGG cell adhesion molecules cams	0.0018	0.035	1.33	122
KEGG ECM receptor interaction	0.0034	0.049	1.36	83
KEGG Wnt signaling pathway	0.0052	0.064	1.27	145
KEGG Hedgehog signaling pathway	0.0057	0.064	1.40	55
KEGG leukocyte transendothelial migration	0.011	0.10	1.28	108

**Correct Table 2.**

**Table 2:** Relevant significantly enriched gene sets associated with the DMPs that differed between aggressive and metastatic PitNETs in the first surgery specimens (upper part of the table) and in the entire cohort (lower part of the table)

Hypermethylated and positive enriched in PC	pval	padj	NES	size
KEGG Cell adhesion molecules CAMS	6.26E-07	5.63E-05	1.65	122
KEGG Axon guidance	6.51E-07	5.63E-05	1.65	122
KEGG Pathways in cancer	1.43E-06	8.24E-05	1.44	313
KEGG Neuroactive ligand receptor interaction	6.39E-06	0.00028	1.44	249
KEGG Focal adhesion	7.16E-05	0.0015	1.44	191
KEGG Adherens junction	8.97E-05	0.0017	1.64	67
KEGG Calcium signaling pathway	0.00025	0.0039	1.40	166
KEGG Leukocyte transendothelial migration	0.0019	0.019	1.43	108
KEGG ECM receptor interaction	0.0019	0.019	1.49	83
KEGG Gap junction	0.0063	0.036	1.40	83

Hypermethylated and positive enriched in PC	pval	padj	NES	size
KEGG Axon guidance	1.60E-06	0.00028	1.53	122
KEGG Calcium signaling pathway	3.22E-06	0.00028	1.44	166
KEGG Neuroactive ligand receptor interaction	0.00010	0.0045	1.31	249
KEGG Regulation of actin cytoskeleton	0.00013	0.0045	1.34	197
KEGG MAPK signaling pathway	0.00030	0.0066	1.29	251
KEGG Cell adhesion molecules cams	0.0018	0.035	1.33	122
KEGG ECM receptor interaction	0.0034	0.049	1.36	83
KEGG Wnt signaling pathway	0.0052	0.064	1.27	145
KEGG Hedgehog signaling pathway	0.0057	0.064	1.40	55
KEGG Leukocyte transendothelial migration	0.011	0.10	1.28	108

