# Supplementary figures and images for: Genome-wide methylation profiling differentiates benign from aggressive and metastatic pituitary neuroendocrine tumors
Source: Acta Neuropathol. 2024 Nov 23;148(1):68. doi: 10.1007/s00401-024-02836-5 (PMC11585505; doi:10.1007/s00401-024-02836-5)

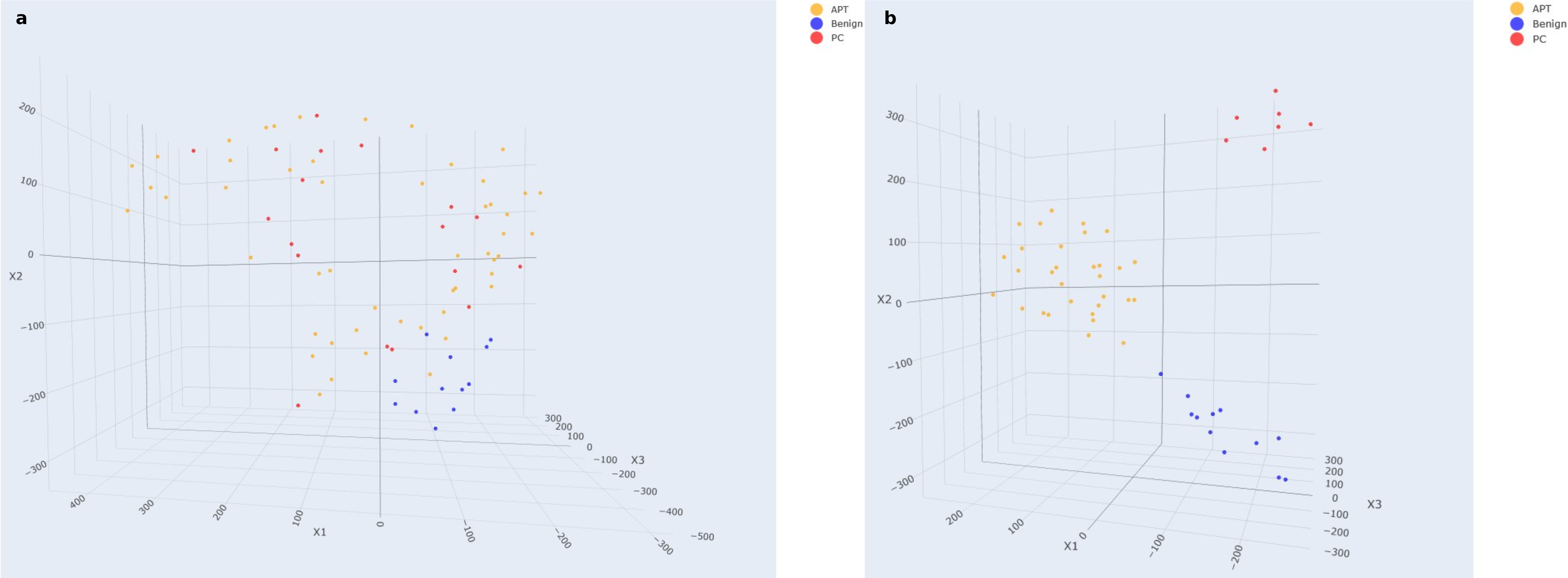

Supplement: Supplementary file 1 — Supplementary file1 (TIFF 5772 KB) [file 401_2024_2836_MOESM1_ESM.tiff]

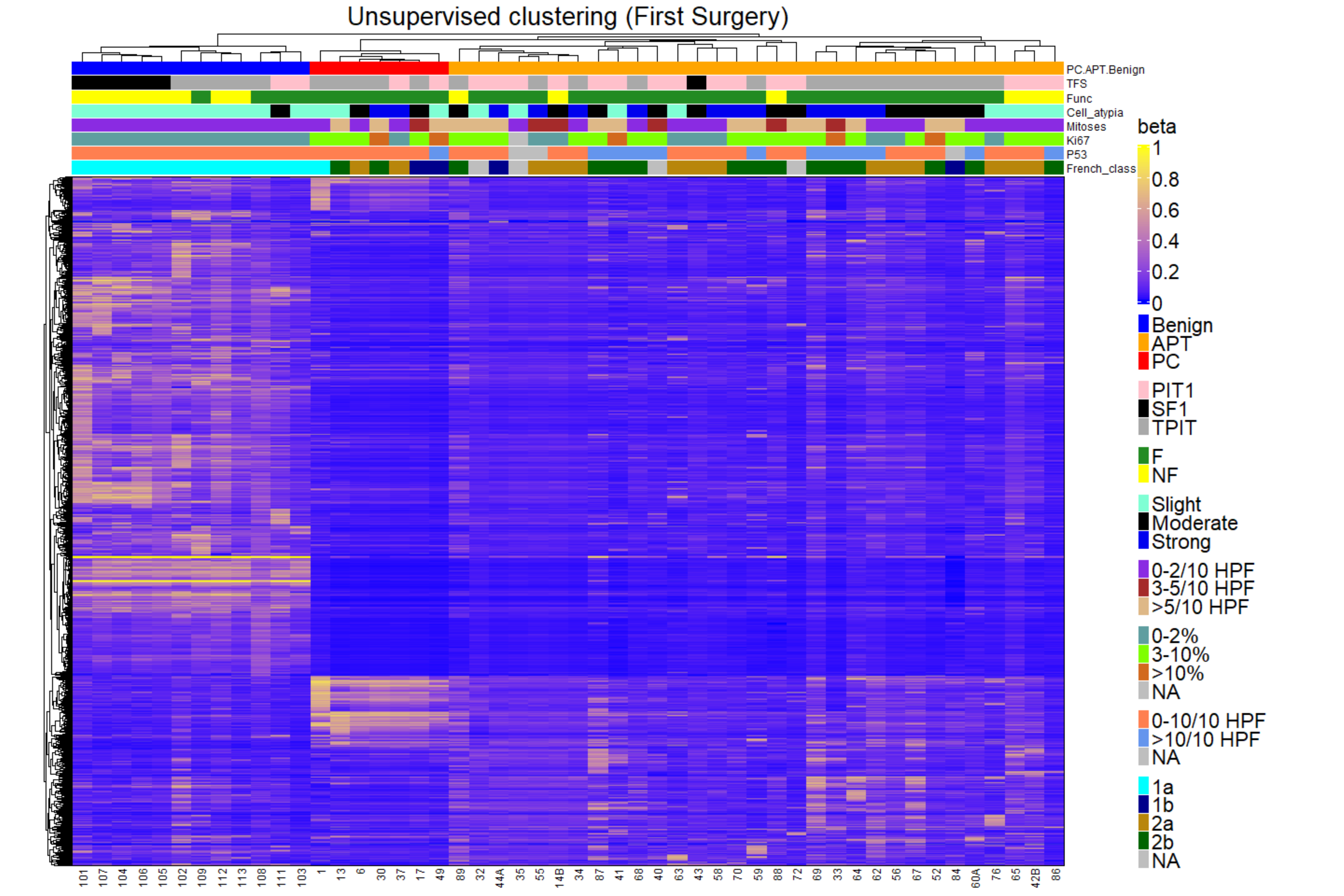

Supplement: Supplementary file 2 — Supplementary file2 (TIFF 3667 KB) [file 401_2024_2836_MOESM2_ESM.tiff]

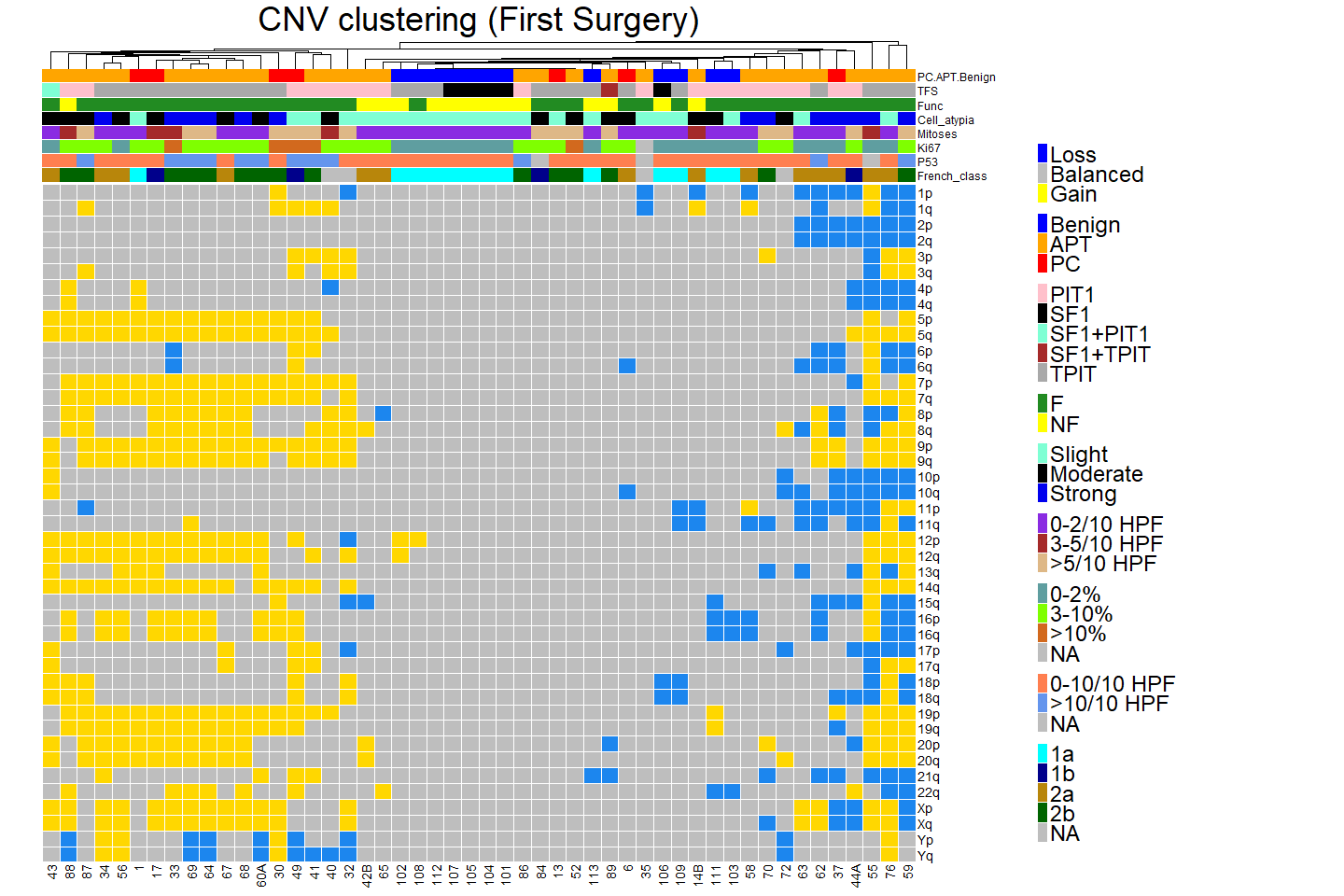

Supplement: Supplementary file 3 — Supplementary file3 (TIFF 2377 KB) [file 401_2024_2836_MOESM3_ESM.tiff]
